# Long term outcome of immunoglobulin A (IgA) nephropathy: A single center experience

**DOI:** 10.1371/journal.pone.0249592

**Published:** 2021-04-08

**Authors:** Rozita Mohd, Nur Ezzaty Mohammad Kazmin, Rizna Abdul Cader, Nordashima Abd Shukor, Yin Ping Wong, Shamsul Azhar Shah, Nurwardah Alfian

**Affiliations:** 1 Department of Medicine, Universiti Kebangsaan Malaysia Medical Centre, Kuala Lumpur, Malaysia; 2 Faculty of Medicine and Health Sciences, Universiti Sains Islam Malaysia, Nilai, Negeri Sembilan, Malaysia; 3 Park City Medical Centre, Kuala Lumpur, Malaysia; 4 Department of Pathology, Faculty of Medicine, Universiti Kebangsaan Malaysia, Kuala Lumpur, Malaysia; 5 Department of Community Health, Faculty of Medicine, Universiti Kebangsaan Malaysia, Kuala Lumpur, Malaysia; University of Glasgow, UNITED KINGDOM

## Abstract

**Introduction:**

IgA nephropathy (IgAN) has a heterogeneous presentation and the progression to end stage renal disease (ESRD) is often influenced by demographics, ethnicity, as well as choice of treatment regimen. In this study, we investigated the long term survival of IgAN patients in our center and the factors affecting it.

**Methods:**

This study included all biopsy-proven IgAN patients with ≥ 1year follow-up. Patients with diabetes mellitus at diagnosis and secondary IgAN were excluded. Medical records were reviewed for demographics, clinical presentation, blood pressure, 24-hour urine protein, serum creatinine, renal biopsy and treatment received. The primary outcome was defined as combined event of 50% estimated glomerular filtration rate (eGFR) reduction or ESRD.

**Results:**

We included 130 (74 females; 56 males) patients of mean age 38.0 ± 14.0 years and median eGFR of 75.2 (interquartile range (IQR) 49.3–101.4) ml/min/1.73m^2^. Eighty-four (64.6%) were hypertensive at presentation, 35 (26.9%) had nephrotic syndrome and 57 (43.8%) had nephrotic range proteinuria (NRP). Median follow-up duration was 7.5 (IQR 4.0–13.0) years. It was noted that 18 (13.8%) developed ESRD and 34 (26.2%) reached the primary outcome. Annual eGFR decline was -2.1 (IQR -5.3 to -0.1) ml/min/1.73m2/year, with median survival of 20 years. Survival rates from the combined event (50% decrease in eGFR or ESRD) at 10, 20 and 30 years were 80%, 53% and 25%, while survival from ESRD were 87%, 73% and 65%, respectively. In the univariate analysis, time-average proteinuria (hazard ratio (HR) = 2.41, 95% CI 1.77–3.30), eGFR <45ml/min/1.73m2 at biopsy (HR = 2.35, 95% CI 1.03–5.32), hypertension (HR = 2.81, 95% CI 1.16–6.80), mean arterial pressure (HR = 1.02, 95% CI 1.01–1.04), tubular atrophy/interstitial fibrosis score (HR = 3.77, 95% CI 1.84–7.73), and cellular/fibrocellular crescent score (HR = 2.44, 95% CI 1.19–5.00) were found to be significant. Whereas only time-average proteinuria (TA-proteinuria) remained as a significant predictor in the multivariate analysis (HR = 2.23, 95% CI 1.57–3.16).

**Conclusion:**

In our cohort, TA-proteinuria was the most important predictor in the progression of IgAN, irrespective of degree of proteinuria at presentation.

## Introduction

IgA nephropathy (IgAN) is the commonest primary glomerulonephritis (GN) worldwide [1]. The diagnosis is based on positive staining of IgA dominant immune complex deposition in the mesangium as noted on renal histopathology and immunofluorescence study [2]. The prevalence of IgA varies widely between continents and ethnic backgrounds. It is reported to be highest in the developed countries in Asia such as Singapore (43.2%) and Japan (31%), followed by some European countries (20–30%) and United States (10–20%) [3]. This variability could be attributed to different healthcare screening policies and biopsy practices as well as genetic and environmental factors [4, 5]. In Malaysia, IgAN (21.7%) is the third commonest cause of primary GN after minimal change disease and focal segmental glomerulosclerosis (FSGS) [6].

IgAN patients have a wide spectrum of clinical presentation, from asymptomatic urinary abnormality, hypertension, nephrotic syndrome to rapidly progressive glomerulonephritis (RPGN) [7, 8]. In the absence of screening programs, it is difficult to pinpoint onset of disease. Thus, many go unnoticed until patients present with significant symptoms and renal impairment. Despite its high prevalence and recent advances in the understanding of the disease, there is still no targeted therapy available for IgAN. Although the anti-proteinuric effect of renin angiotensin-aldosterone system (RAAS) inhibitors has been shown to be impressive in certain groups of patients [9], the treatment choice and benefits of immunosuppression in patients with highest risk of progression is still controversial and associated with significant side effects [10–13].

Recent studies have found that IgAN has a variable natural progression as less than 10% up to 30% of patients progress to end stage renal disease (ESRD) within 10 years of renal biopsy [14–16]. Patients of Pacific Asian origin appear to have faster rates of renal function decline and poorer kidney outcomes [5, 17]. Extensive efforts have been made in the recent decade to explore predictors that could predict long term outcomes of IgAN progression. These include clinical, biochemical, and histological risk factors, both at diagnosis as well as during follow up. Cross sectional clinical data at biopsy and follow up such as proteinuria of > 1g per day, decrease in eGFR and presence of hypertension were found to be associated with a faster rate of progression to ESRD [12, 18]. The recently developed Oxford classification of IgAN identified five renal histological lesions, namely mesangial hypercellularity (M), endocapillary hypercellularity (E), segmental sclerosis (S), tubular atrophy (T) and crescents (C). These constitute the MEST-C Score to independently predict renal outcome [19]. Some studies have demonstrated that during a two-year follow up duration, histological lesions predicted renal outcomes as equally efficacious as clinical parameters [20].

To date, the long term renal outcomes of IgAN have not been well described in the Malaysian multi-ethnic population. Furthermore, based on The Malaysian Registry of Renal Biopsy (MRRB) data, Malaysian IgA patients tend to present with a more severe form of clinical presentation [6]. Hence, this study aimed to investigate the clinicopathological characteristics, treatment, and long-term renal outcomes of IgAN patients diagnosed in our center.

## Materials and method

This study was approved by the Medical Ethics Committee of Universiti Kebangsaan Malaysia (project code: FF-2019-305) and was in accordance with the Declaration of Helsinki. Informed consent was waived by the ethics committee because of the retrospective nature of the study and the analysis used anonymous data. This study included all IgAN patients diagnosed and treated in Hospital Universiti Kebangsaan Malaysia between January 01, 1986 and August 31, 2020. Access to paper and electronic medical records were obtained from Medical Record Office of Hospital Universiti Kebangsaan Malaysia and the biopsy samples were retrieved from Pathology Department of Hospital Universiti Kebangsaan Malaysia. The medical records and the biopsy samples obtained were accessed and reviewed between June 01, 2019 and August 31, 2020.

Biopsy-proven IgAN patients with minimum of 1 year follow up were included in the study. We excluded patients who had diabetes mellitus at diagnosis as well as secondary causes of IgAN such as Henoch-Schonlein purpura (HSP), chronic liver disease, systemic lupus erythematosus (SLE) and other autoimmune disorders.

Clinical characteristics and baseline demographic data were collected which included age, gender, ethnicity, systolic blood pressure (SBP), and diastolic blood pressure (DBP). Initial clinical presentation, comorbidities as well as treatment given 6 months prior to renal biopsy were obtained.

Symptomatic presentation was categorized into predominant clinical syndromes of either Nephrotic Syndrome, Nephritic Syndrome or Gross Hematuria. Nephrotic syndrome is defined as clinical sign of edema in the presence of heavy proteinuria ≥3.0 g/day and/or hypoalbuminemia [21]. Nephritic Syndrome is defined as mild to moderate degree of proteinuria (<3.0g/day) with hematuria, increase in serum creatinine, and/or hypertension [22]. Gross Hematuria is defined as urine that is visibly pink, tea, cola-colored or red. Asymptomatic presentation is defined as incidental findings of urinary abnormality, abnormal creatinine and or hypertension in a well patient during a medical check-up or follow up unrelated to IgAN.

Hypertension was defined as systolic blood pressure (SBP) ≥ 140 mmHg or diastolic blood pressure (DBP) ≥ 90 mmHg, reported history of hypertension or use of antihypertensive medications. Mean arterial pressure (MAP) was defined as the sum of diastolic blood pressure (DBP) and one-third of the pulse pressure (SBP-DBP).

Laboratory data collected at renal biopsy and during follow up included serum creatinine and proteinuria expressed in gram per 24 hours (g/day). Nephrotic range proteinuria (NRP) is defined as laboratory finding with proteinuria of ≥3.0g/day irrespective of edema or serum albumin levels. The eGFR was estimated using the 4 variable Modification of Diet in Renal Disease (MDRD) formula [23]. Average proteinuria during follow up was expressed as time-average proteinuria (TA-proteinuria); this represented the average proteinuria for each follow up period.

RAAS blocker use was defined as treatment with either angiotensin-converting enzyme inhibitors, angiotensin receptor blockers, spironolactone, or any of the mentioned combination. Immunosuppressive treatment was recorded based on intention to treat regardless of dose and duration. Any treatment given within 1 year after renal biopsy was considered as the initial treatment.

The available renal biopsies were reviewed by 2 independent histopathologists and the findings were graded according to the Oxford MEST-C Classification: mesangial hypercellularity M0 or M1 (< or ≥ 50% of glomeruli had more than three cells per mesangial area); endocapillary hypercellularity E0 (absent) or E1 (present); segmental glomerulosclerosis S0 (absent) or S1 (present); tubular atrophy/interstitial fibrosis score T0 (0–25%), T1 (26–50%) or T2 (>50%) and cellular or fibrocellular crescent score C0 (no crescent), C1 (0–25%) or C2 (≥25%) of glomeruli present. Any discrepancies with the scoring were reviewed and discussed together until a consensus was achieved.

### Outcome measurements

The primary outcome was defined as a combined event of 50% decrease in the eGFR (compared to baseline at renal biopsy) or ESRD. Secondary outcomes analyzed separately were ESRD, 50% decrease in eGFR and the rate of annual decline in eGFR.

### Statistical analysis

Data were analyzed using SPSS software (version 26). All categorical variables were presented as frequencies and percentages. For numerical variables, normally distributed data were expressed as mean ± standard deviation (S.D) whereas those without normal distribution were expressed as median and interquartile range (IQR).

Next, a comparison between the outcome groups was done using Chi-Square test for dichotomous variables, student t-test for parametric continuous variables and Mann-Whitney test for non-parametric variables.

Kaplan-Meier analyses were used to describe the time of event-free survival. Survival differences between groups were tested with a log-rank procedure. Univariate and multivariate Cox regression analysis were used to evaluate the risk of deterioration to 50% decrease in eGFR or ESRD. Multivariate analysis included the factors that differed significantly in the univariate analysis. The results of the univariate and multivariate analyses were expressed as hazard ratios (HRs) with 95% confidence intervals (CIs).

The renal function decline rate was calculated to represent the average of each follow up period’s mean decline in eGFR. All p values were two-tailed, and values less than 0.05 were considered as statistically significant.

## Results

Between 1986 to 2019, 198 patients were diagnosed with biopsy proven primary IgA nephropathy at our center. After exclusion of 68 patients with missing data, we included 130 patients with a minimum follow up duration of 1 year for final analysis and the baseline characteristics are shown in Table 1. Majority of the patients were Malay (54.6%), followed by Chinese (39.2%) and a small percentage of Indians. Most of them (64.6%) were hypertensive at presentation with a third (21.5%) being newly diagnosed. Although asymptomatic presentation (58.5%) was the commonest in this cohort, 26.9% of them presented with nephrotic syndrome. As for degree of proteinuria, among 130 patients studied, 84.6% of patients at renal biopsy had urine protein ≥ 1 g per day and 43.8% were within nephrotic range, with urine protein of ≥ 3 g per day (nephrotic range proteinuria, NRP). The median eGFR was 75.2 (IQR: 49.3 to 101.4 ml/min per 1.73m^2^) and 12.3% of them had eGFR <30 ml/min per 1.73m^2^ at the point of diagnosis.

**Table 1:** Baseline characteristics of patients with IgA nephropathy.

Baseline Characteristics	n = 130 (%)	Mean ± SD or Median (IQR)
Age (years)		38.0 ± 14.0
Female (%)	74 (56.9)	
***Ethnicity (%)***		
Malay	71 (54.6)	
Chinese	51 (39.2)	
Indian	2 (1.5)	
Others	6 (4.6)	
Hypertensive subjects (%)	84 (64.6)	
SBP (mm Hg)		137 (126 to 154)
DBP (mm Hg)		81 (74 to 95)
MAP (mm Hg)		99 (91 to 113)
***Clinical Presentation***		
Asymptomatic (%)	76 (58.5)	
Urinary abnormality	76 (58.5)	
Abnormal creatinine	18 (13.8)	
Newly diagnosed hypertension	28 (21.5)	
Symptomatic (%)	54 (41.5)	
Nephrotic Syndrome	35 (26.9)	
Nephritic Syndrome	11 (8.5)	
Gross Hematuria	8 (6.2)	
***Laboratory test***		
Proteinuria (g/day)		2.4 (1.4 to 5.1)
**Proteinuria g/day (%)**		
<0.5	6 (4.6)	
≥0.5 <1.0	14 (10.8)	
≥1.0 <3.0	53 (40.8)	
≥ 3.0	57 (43.8)	
eGFR (ml/min per 1.73^2^)		75.2 (49.3 to 101.4)
**CKD Stage (%)**		
I	47 (36.2)	
II	36 (27.7)	
III	31 (23.8)	
IV	15 (11.5)	
V	1 (0.8)	
***Treatment before renal biopsy (%)***	54 (41.5)	
Immunosuppressive treatment (%)	11 (8.5)	
RAAS Blockade (%)	30 (23.1)	

SBP: systolic blood pressure, DBP: diastolic blood pressure, MAP: mean arterial pressure, eGFR: estimated glomerular filtration rate, CKD: Chronic kidney disease, RAAS: renin-angiotensin-aldosterone system.

Symptomatic refers to predominant clinical presenting syndrome, based on symptoms as well as laboratory criteria, i.e., Nephrotic Syndrome, Nephritic Syndrome, or Gross Hematuria.

Results were expressed as mean ± SD, median (interquartile range), or percentage.

Patients were followed up with a median duration of 7.5 (IQR 4.0 to 13.0 years) and the details are shown in Table 2. Despite the median proteinuria at biopsy of 2.4 (IQR: 1.4 to 5.1 g/day), the time-average proteinuria (TA-proteinuria) during follow up was only 0.7 (IQR: 0.4 to 1.4 g/day).

**Table 2:** Follow-up data and clinical outcomes of patients with IgA nephropathy.

Follow up data	n = 130 (%)	Mean ± (SD) or Median (IQR)
Duration of follow up; median (years)		7.5 (4.0 to 13.0)
TA Proteinuria (g/day)		0.7 (0.4 to 1.4)
**TA Proteinuria (%)**		
<0.5	36 (27.7)	
≥0.5 <1.0	42 (32.3)	
≥1.0 <1.5	20 (15.4)	
≥1.5 <2.0	13 (10)	
≥ 2.0 <2.5	7 (5.4)	
≥ 2.5 <3.0	5 (3.8)	
≥ 3.0	7 (5.4)	
***Immunosuppression***^◊^		
Corticosteroid (%)	83 (63.8)	
Cyclophosphamide (%)	35 (26.9)	
MMF (%)	16 (12.3)	
Calcineurin inhibitors (%)	33 (25.4)	
Azathioprine (%)	17 (13.1)	
***Non-immunosuppression***^◊^		
RAAS Blockade (%)	122 (93.8)	
Fish Oil (%)	82 (63.1)	
Calcium Channel Blocker (%)	73 (56.2)	
Antiplatelet agents (%)	13 (10)	
***Long term Complication***^◊^***(%)***		
Diabetes Mellitus	20 (15.4)	
Malignancy	5 (3.8)	
**Renal Biopsy Oxford MEST-C Scoring**		
M1	65 (50)	
E1	13 (10)	
S1	88 (67.7)	
T1	22 (16.9)	
T2	11 (8.5)	
C1	27 (20.8)	
C2	7 (5.4)	
**Clinical Outcome Data**		
Rate of decline in renal function (ml/min/1.73m^2^/year)		- 2.1 (-5.3 to -0.1)
ESRD (<15ml/min/1.73m^2^)	18 (13.8)	
50% decrease in eGFR	33 (25.4)	
50% decrease in eGFR or ESRD	34 (26.2)	

TA: time average, MMF: Mycophenolate Mofetil, RAAS: renin-angiotensin-aldosterone system, eGFR: estimated glomerular filtration rate, ESRD: End Stage Renal Disease.

^◊^Each patient can have more than one treatment group and long-term complications. Results are expressed as mean ± SD, median (interquartile range), or percentage.

Notably, a high percentage of RAAS blockers were used (93.8%) in this study group of patients. The use of immunosuppression was also high with use of corticosteroids (63.8%), followed by cyclophosphamide (26.9%) and calcineurin inhibitors (25.4%).

Histological examination revealed that 67.7% of the renal biopsies showed segmental glomerulosclerosis (S1) with only 10% demonstrating endocapillary hypercellularity (E1) lesion. Cellular/fibrocellular crescents were present in 26.2% of the biopsies. Of the Oxford MEST-C Scores, only tubular atrophy/interstitial fibrosis (T) score was positively associated with a higher MAP of 107mmHg (p = 0.005) and a lower eGFR at renal biopsy (< 45.1 ml/min per 1.73m^2^) (p<0.001) (Table 3).

**Table 3:** Comparison of clinical parameters at renal biopsy against MEST-C scores.

	MAP (mmHg)	p value	eGFR (ml/min/1.73m^2^)	p value	Proteinuria (g/day)	p value
Mesangial proliferation						
M0	99 (90 to 112)	0.510	78.9 (48.9 to 115.3)	0.581	2.8 (1.4 to 5.2)	0.667
M1	101 (92 to 116)		74.5(47.4 to 96.3)		2.1 (1.5 to 5.1)	
Endocapillary hypercellularity						
E0	99 (91 to 113)	0.753	77.7 (52.4 to 101.7)	0.172	2.4 (1.4 to 5.1)	0.704
E1	103 (85 to 120)		49.9 (28.5 to 100.1)		2.7 (1.5 to 4.9)	
Segmental glomerulosclerosis						
S0	103 (89 to 114)	0.582	76.3 (53.1 to 107.7)	0.954	2.5 (1.2 to 5.2)	0.846
S1	98 (92 to 113)		75.0 (45.9 to 100.7)		2.4 (1.5 to 5.1)	
Tubular atrophy/interstitial fibrosis						
T0	97 (89 to 111)	**0.005**	83.1 (59.8 to 117.2)	**<0.001**	2.4 (1.3 to 4.8)	0.594
T1-2	107 (95 to 120)		45.1 (28.8 to 69.1)		2.4 (1.6 to 5.5)	
Crescents						
C0	99 (92 to 111)	0.302	78.3 (53.4 to 113.0)	0.082	2.4 (1.4 to 5.1)	0.865
C1-2	104 (89 to 121)		64.1 (29.7 to 92.3)		2.6 (1.5 to 5.2)	

MAP: mean arterial pressure, eGFR: estimated glomerular filtration rate.

The median rate of eGFR decline was -2.1 (IQR -5.3 to -0.1 ml/min per 1.73m^2^/year). Thirty-four patients (26.2%) reached the primary outcome of 50% decrease in eGFR or ESRD with median survival of 20 years.

**Fig 1:**
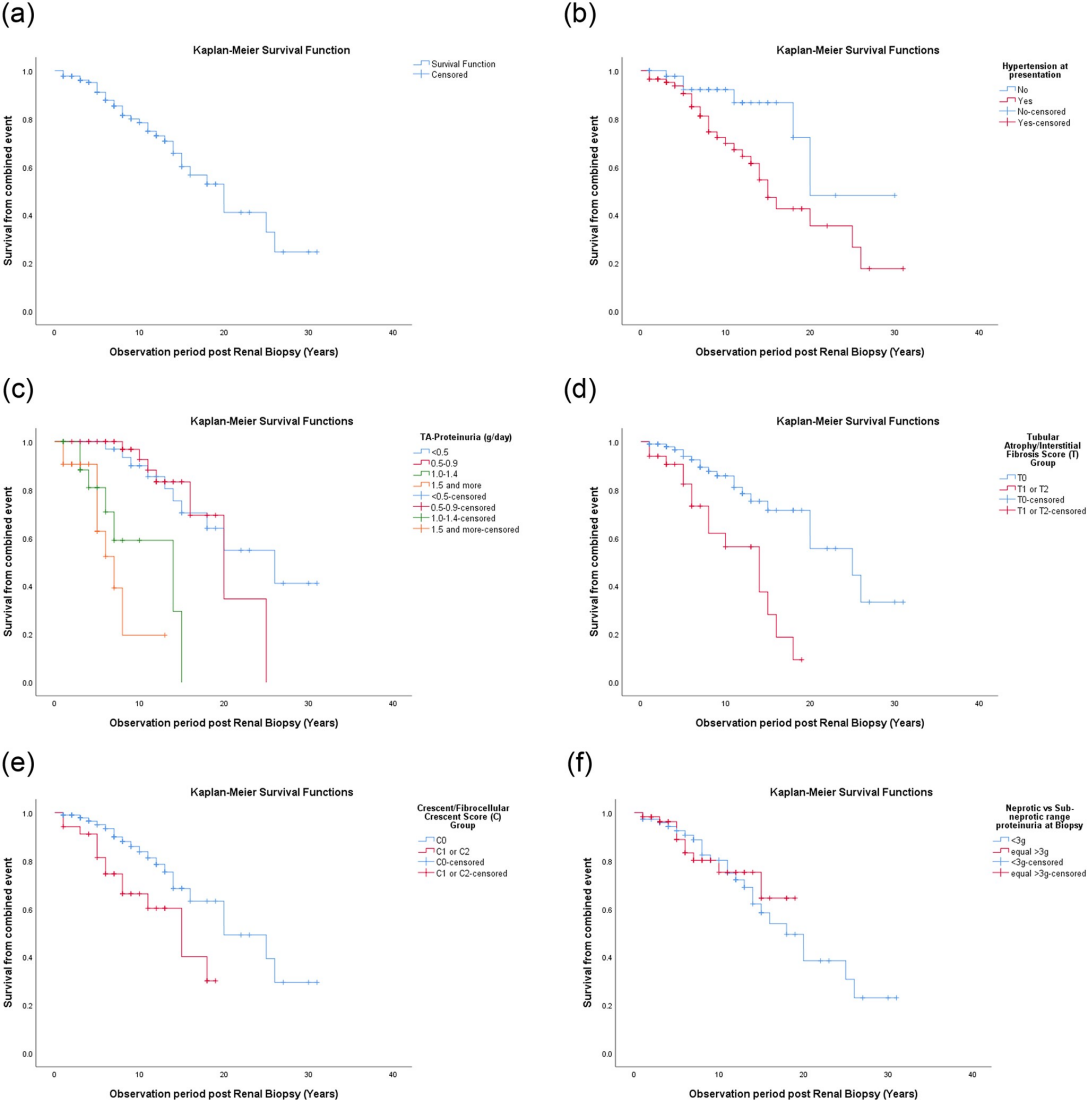
Primary endpoint analysis, i.e., time to first occurrence of combined event of 50% decrease in eGFR or ESRD. (a) Kaplan-Meier curves showing survival from combined event in the entire cohort, categorized by, (b) presence of hypertension at clinical presentation (c) degree of TA-proteinuria, (d) tubular atrophy/interstitial fibrosis (T) score (e) cellular/fibrocellular crescent (C) score and (f) nephrotic range proteinuria (NRP) with sub-nephrotic range proteinuria at biopsy.

**Fig 2:**
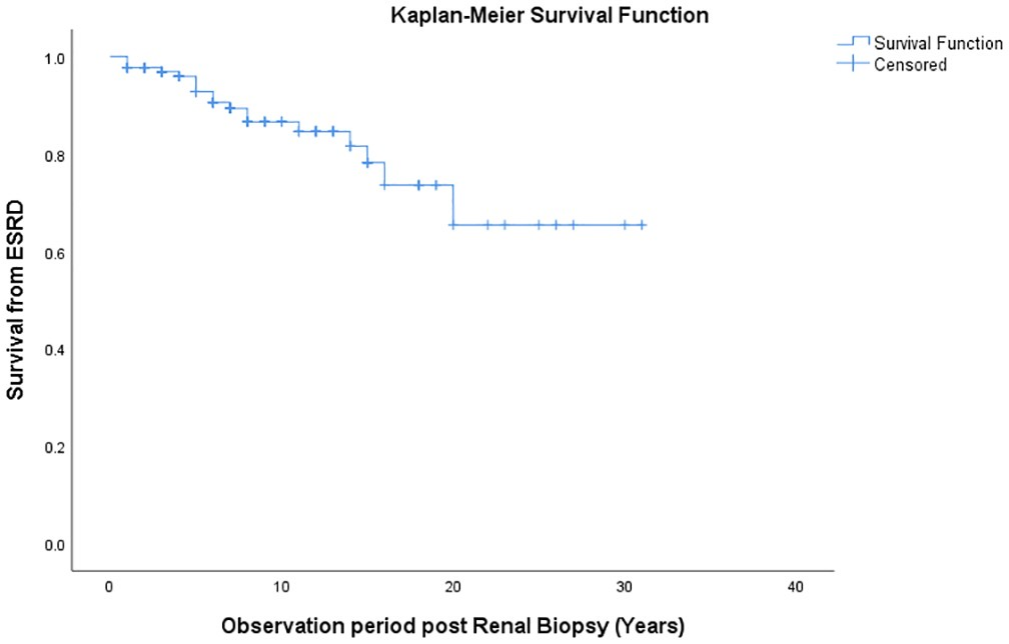
Secondary endpoint analysis, i.e., time to development of ESRD. Kaplan-Meier curve showing the survival from ESRD in the entire cohort.

Based on the Kaplan-Meier curves, survival rates without combined event of the entire cohort at 10, 20 and 30 years were 80%, 53% and 25%, respectively (Fig 1a). The survival without ESRD was 87%, 73% and 65%, respectively (Fig 2). The Kaplan Mayer curves showed significant differences in survival rate based on the presence of hypertension at presentation (p = 0.016) (Fig 1b), degree of TA-Proteinuria (p <0.001) (Fig 1c), T (p <0.001) (Fig 1d) and C scores (p = 0.011) (Fig 1e). There was no significant survival difference between patients presenting with nephrotic range proteinuria (NRP) and those with sub-nephrotic range proteinuria (p = 0.785) (Fig 1f).

### Correlations between clinical, laboratory and histopathology predictors with outcomes

A risk factor assessment for the primary outcome of 50% decrease in eGFR or ESRD was performed and summarized in Table 4. In the univariate analysis, survival from the combined event was predominantly affected by degree of TA-proteinuria, even by TA-proteinuria as low as ≥ 0.5g per day. The eGFR at renal biopsy was a significant predictor for survival from combined event beyond stage 3b (eGFR <45 ml/min per 1.73m^2^) while the clinical factors of hypertension at presentation and MAP at renal biopsy were also statistically significant. Histopathologically, only tubular atrophy/interstitial fibrosis score and cellular/fibrocellular crescent score were found to serve as significant predictors of survival from the combined event in this cohort. The only predictor that remained significant at multivariate analysis was TA-proteinuria.

**Table 4:** Correlations between clinical, laboratory and pathology with renal outcomes.

Variable	Survival from decrease in eGFR or ESRD (combined event)			
	Univariate Hazard Ratio (95% CI)		Multivariate Hazard Ratio (95% CI)^a^	
**Clinical Presentation**				
With Hypertension	2.81 (1.16–6.80)	**p = 0.022**	1.95 (0.71–5.37)	p = 0.196
Without Hypertension	1		1	
**Mean Arterial Pressure at Renal Biopsy**	1.02 (1.01–1.04)	**p = 0.001**	1.01 (0.99–1.03)	p = 0.504
**24 Urine Protein at Renal Biopsy**	0.99 (0.89–1.12)	p = 0.956		
**eGFR at Renal Biopsy**	0.99 (0.98–1.00)	p = 0.117		
**eGFR groups at Renal Biopsy**				
< 60 ml/min/1.73m^2^	1.88 (0.93–3.79)	p = 0.078		
≥ 60 ml/min/1.73m^2^	1			
< 45 ml/min/1.73m^2^	2.35 (1.03–5.32)	**p = 0.042**	0.53 (0.18–1.53)	p = 0.237
≥ 45 ml/min/1.73m^2^	1		1	
< 30 ml/min/1.73m^2^	5.80 (2.39–14.06)	**p<0.001**		
≥ 30 ml/min/1.73m^2^	1			
**TA-proteinuria**	2.41 (1.77–3.30)	**p<0.001**	2.23 (1.57–3.16)	**p<0.001**
**TA-proteinuria groups**				
≥ 0.5 g/24 Hours	2.57 (1.15–5.73)	**p = 0.021**		
< 0.5/24 Hours	1			
≥ 1 g/24 Hours	9.91 (4.39–22.39)	**p<0.001**		
< 1 g/24 Hours	1			
≥ 3 g/24 Hours	21.88 (6.0–79.81)	**p<0.001**		
< 3 g/24 Hours	1			
**Histological Scoring**				
***Mesangial hypercellularity***				
M1	0.66 (0.33–1.31)	p = 0.235		
M0	1			
*Endocapillary hypercellularity*				
E1	0.96 (0.29–3.16)	p = 0.948		
E0	1			
***Segmental glomerulosclerosis***				
S1	1.69 (0.78–3.68)	p = 0.184		
S0	1			
***Tubular atrophy/ interstitial fibrosis***				
T1 or T2	3.77 (1.84–7.73)	**p<0.001**	2.07 (0.85–5.03)	p = 0.109
T0	1		1	
***Cellular or fibrocellular crescents***				
C1 or C2	2.44 (1.19–5.00)	**p = 0.015**	1.95 (0.83–4.57)	p = 0.123
C0	1		1	

CI: confidence interval, eGFR: estimated glomerular filtration rate, ESRD: End Stage Renal Disease.

^a^ Multivariate model are adjusted for presence of Hypertension at clinical presentation, MAP at Renal Biopsy, eGFR 45ml/min/1.73m2, TA-proteinuria, T & C Score.

### Subgroup analysis with nephrotic range proteinuria at renal biopsy

Fifty-seven patients (43.8%) presented with nephrotic range proteinuria (NRP) in this group, and their clinicopathological characteristics are compared in Table 5. It was found that more patients with NRP presented with symptoms (66.7%) with median range proteinuria of 5.6 (IQR: 3.9 to 7.6 g/day) (p<0.001). Henceforth, they were treated with more immunosuppressive agents (p<0.05). The difference between RAAS blockers used at renal biopsy was no longer statistically significant at 1 year and throughout follow up. Despite the high median urine protein at diagnosis, the median TA-proteinuria was 1.26 (IQR 0.69 to 2.22 g/day). Interestingly, the frequency of patients who developed a combined event of 50% decrease in eGFR or ESRD was significantly lower in the NRP group (17.5%) compared to that of the sub-nephrotic group (32.9%) (p = 0.048). The lower rate of 50% decrease in eGFR in these NRP patients (p = 0.026) contributed significantly to this.

**Table 5:** Clinical, laboratory and histological characteristics of patients with IgA nephropathy presenting with nephrotic range proteinuria and sub-nephrotic proteinuria.

	Sub-nephrotic range proteinuria (n = 73)	Nephrotic range proteinuria (n = 57)	*p*
***Clinical Data***			
Age (Mean;SD)	35.8 ±12.5	40.7 ±15.4	0.054
Female: Male (n)	44: 29	30:27	0.383
Malay (%)	39 (53.4)	32 (56.1)	0.758
Hypertension (%)	49 (67.1)	35 (61.4)	0.499
SBP (mm Hg) (IQR)	138 (126 to 153)	137 (126 to 160)	0.899
DBP (mm Hg) (IQR)	83 (73 to 98)	80 (75 to 94)	0.974
MAP (mmHg) (IQR)	101 (90 to 116)	98 (93 to 113)	0.931
Symptomatic Presentation (%)	18 (24.7)	36 (63.2)	**<0.001**
***Laboratory Data at Biopsy***			
eGFR (ml/min/1.73m^2^)	82.0 (56.0 to100.9)	65.4(36.2 to 104.4)	0.089
Proteinuria (g/24 Hours) (IQR)	1.5 (0.9 to 2.1)	5.6 (3.9 to7.6)	**<0.001**
***MEST-C Lesion (%)***			
M1 (%)	39 (53.4)	26 (45.6)	0.377
E1(%)	7 (9.6)	6 (10.5)	0.860
S1(%)	48 (65.6)	40 (70.2)	0.593
T1 or T2(%)	19 (26.0)	14 (24.6)	0.849
C1 or C2(%)	18 (24.7)	16 (28.1)	0.660
***Follow up Data***			
Duration of follow up (years)	9 (4 to14)	6 (3.5 to 10.5)	**0.033**
***Treatment before Renal Biopsy***			
RAAS (%)	17 (23.3)	13 (22.8)	0.949
Steroid (%)	2 (2.7)	9 (15.8)	**0.008**
***Treatment at Renal Biopsy***			
RAAS Blocker (%)	62 (84.9)	35 (61.4)	**0.002**
Any Immunosuppression (%)	11 (15.1)	41 (71.9)	**<0.001**
***Treatment 1-year post biopsy***			
RAAS Blocker (%)	65 (89.0)	49 (86.0)	0.596
Any Immunosuppression (%)	16 (21.9)	47 (82.5)	**<0.001**
***Treatment (overall follow up)***			
RAAS Blocker (%)	69 (94.5)	53 (93.0)	0.729
Any Immunosuppression (%)	33 (45.2)	51 (89.5)	**<0.001**
Corticosteroid	32 (43.8)	51 (89.5)	**<0.001**
Cyclophosphamide	7 (9.6)	28 (49.1)	**<0.001**
Calcineurin Inhibitors	11 (15.1)	22 (38.6)	**0.002**
TA-Proteinuria (g/day)	0.50 (0.29 to 0.90)	1.26 (0.69 to 2.22)	**<0.001**
***Outcome***			
Rate of decline in eGFR (ml/min/1.73m^2^/year)	-2.1 (-5.6 to -0.7)	-2.6 (-5.1 to 1.5)	0.335
50% decrease eGFR (%)	24 (32.9)	9 (15.8)	**0.026**
ESRD (<15ml/min/ min/1.73m^2^)	11 (15.1)	7 (12.3)	0.648
50% decrease in eGFR or ESRD (%)	24 (32.9)	10 (17.5)	**0.048**

SBP: systolic blood pressure, DBP: diastolic blood pressure, MAP: mean arterial pressure, TA: time average, MMF: Mycophenolate Mofetil, RAAS: renin-angiotensin-aldosterone system, eGFR: estimated glomerular filtration rate, ESRD: End Stage Renal Disease.

## Discussion

The present study was the first IgA study in which the majority of patients were from the Malay ethnic group (54.6%), followed by Chinese ethnicity (39.2%), indigenous ethnicity (4.6%) and Indian ethnicity (1.5%). The lower recruitment rate of Indian patients in this study could be explained by the demographic population surrounding our hospital locality. Interestingly, compared to the most recently reported studies [24, 25], our group of patients were predominantly female (56.7%) and this concurred with our country’s National Renal Biopsy Registry Report 2017 [6]. Most Western cohorts had reported more male patients with IgAN [20, 26, 27], while studies in Asia reported an almost equal ratio of male to female patients (1:1) [28–30]. Although this variation could reflect the possible differences in the underlying pathogenic process, it could also suggest a higher nationwide urinary screening rate during pregnancy in our country.

Compared to other Asian and European countries, our patients were biopsied at an older age [24, 25, 28, 30]. We believed that most of our patients were diagnosed at a later stage of the disease due to our biopsy practices that defer renal biopsy unless the patient has persistent proteinuria of ≥ 1 g/24 hours or signs of renal impairment. The younger age of diagnosis at other regions could also be related to the presence of nationwide urinary screening prior to military service or employment [4].

Our IgA cohort had a significant higher percentage of hypertension (64.6%) which was almost double than the reported rates in other Asian countries which ranged between 30–38.7% [29–32]. This could be explained by the observation that our patients presented at a much later stage, only when they were either symptomatic or being screened opportunistically during pregnancy; whereas in other countries like in Japan, school age children are routinely screened for proteinuria [4]. Essential hypertension is also known to be more prevalent at older age.

Our cohorts also displayed alarmingly higher percentages of NRP (43.8%) and nephrotic syndrome (26.9%) as compared to that in China with a prevalence of nephrotic syndrome at 14.7% [33], 10.1% in Korea [34], and 3.3% in Japan [35]. The findings are believed to be consistent nationwide, according to the Malaysian Registry of Renal Biopsy (MRRB) 2017 [6]. Although the likelihood of selection bias cannot be eliminated entirely, the possibility of the unique genetic or environmental factors leading to such findings are yet to be explored.

Interestingly, despite a higher 24-hour urine protein at presentation with median of 2.4 g/day (IQR 1.4–5.1), our patients’ TA-proteinuria was only 0.73 g/day (IQR 0.42–1.43), which was comparable to most studies [24, 36]. Furthermore, we found that 24-hour urine protein at diagnosis was not a significant predictor of renal outcomes (p = 0.956). Instead, TA-proteinuria was found to be the strongest and the only predictor that remained significant with multivariate analysis (HR 2.23, CI 1.57–3.16, p<0.001).

While many studies reported that the 24-hour urine protein at diagnosis was useful to predict renal outcomes [37, 38], some studies did not [14, 39]. Our findings concurred with many studies that the average proteinuria during follow up had a higher predictive value than the degree of proteinuria at diagnosis [14, 29, 40–42]. The present study also proved that it was possible to reduce the average proteinuria to lower levels with appropriate treatment, despite the relatively high degree of urine protein at presentation.

In the present study, the NRP group is less likely to develop 50% decrease in eGFR although there was no significant difference in the survival from the combined event compared to the sub-nephrotic group (Fig 1f). There are few possible reasons for this observation. Firstly, the NRP patients were mostly symptomatic. Therefore, they were treated much earlier and more aggressively with significantly higher usage of immunosuppressant drugs that resulted in a higher percentage reduction of proteinuria achieved by the NRP group (-76.8%) compared to the sub-nephrotic proteinuria group (-66.7%) relative to baseline. In our study, those who presented with NRP were treated with pulse intravenous methylprednisolone followed by intravenous cyclophosphamide 2 weekly for a total of 3 months. Treatment duration was extended to 6 months in patients with rapidly progressive glomerulonephritis (RPGN). Many patients from this group would later require steroid sparing agents in the form of calcineurin inhibitors, mycophenolate mofetil (MMF) or azathioprine.

Proteinuria reduction has been proposed as surrogate end point in the trial of IgAN [43]. Trial-level analysis from 13 controlled trials have showed the association between the percentage reduction of proteinuria and treatment effects on composite outcome of the time to doubling of serum creatinine level, ESRD, or death [43]. Many studies had also demonstrated that a higher use of immunosuppression was associated with a higher degree of proteinuria reduction [13, 40, 44, 45].

On the other hand, a study by Rauen et al. showed initial proteinuria reduction with immunosuppression in patients with moderate proteinuria (0.75–3.5g/day) [10], but passive follow up of the cohort failed to show a significant difference in the primary outcome [46]. At the same time, it was found that 3-fold fewer patients with full remission in the original trial finally went on to reach the primary outcome in the follow up study [46]. This, together with our study findings as well as studies mentioned earlier, suggests that sustained proteinuria reduction (as reflected by lower TA-Proteinuria in this study) has a more important role than transient proteinuria reduction in preventing CKD progression in IgA Nephropathy.

Compared to most studies [24, 32, 47–49], our cohort were treated with very high rates (93.8%) of RAAS blockers. Despite the initial difference at biopsy, the subsequent use of RAAS blockers was comparable between the NRP and the sub nephrotic groups. This initial difference could also be explained by a higher percentage of patients presenting with acute kidney injury (AKI), hence the delay in RAAS introduction to the NRP group of patients.

Similar reasons could clarify the significantly lower incidence of 50% eGFR decrease in the NRP patients. We believed that despite the apparently lower eGFR in patients with NRP at biopsy, their actual eGFR could have been higher but it was masked due to AKI as a result of severe nephrosis or diuretics. Hence, once the eGFR recovers to the true baseline, even with progression, this would result in a lower percentage of eGFR decrease compared to eGFR at biopsy.

Finally, in our patients, the 10-year survival from the combined event (50% decrease in eGFR or ESRD) was noted to be 80%. This is similar to reports worldwide which range between 80% and 85% [37]. However, only few studies have described long term renal survival beyond 10 years [32, 50] and majority of the studies have used the ESRD outcome alone to estimate renal survival. In our study, the 10, 20 and 30-year renal survival without ESRD was 87%, 73% and 65%, respectively. 20-year renal survival without ESRD was reported to be 66.6–72.5% in Japan [28, 32], 70.8% in Korea [30] and 64% in China [29]. On the other hand, the 30-year survival without ESRD was reported to be 67.3% in Korea [30], and 50.3% in Japanese population [28]. This shows that despite our patients presenting with more severe features and the differences in biopsy practice, the long-term renal outcome was comparable to reports in other populations.

There are a few limitations of this study that must be recognized. Firstly, the data was obtained retrospectively and as with all long-term studies, missing data were unavoidable. Secondly, despite the data on treatment allocation, patient’s compliance as well as possible adverse effects of the medications were largely unknown. At the same time, despite our best efforts, there is the possibility of unmeasured variables confounding the renal outcomes, which include long term side effects of immunosuppressive therapy itself. Lastly, we were unable to ascertain if the unexpected favorable outcome of NRP patients were due to inclusion of IgA nephropathy with superimposed minimal change disease (IgA-MCD), as electron microscopic examination of the biopsy samples were not performed. Having said that, reports have shown that this entity is still quite rare [51, 52]. At the same time in this study, no significant MEST-C score difference was noted between the sub-nephrotic and NRP group of patients.

## Conclusion

In this study, TA-proteinuria was found to be the most important predictor in the progression of IgAN, irrespective of the degree of proteinuria at presentation.

## Supporting information

S1 File IgA analysis data simplified. (SAV)
